# An electrophysiological correlate of sleep in a shark

**DOI:** 10.1002/jez.2846

**Published:** 2024-07-03

**Authors:** John A. Lesku, Paul‐Antoine Libourel, Michael L. Kelly, Jan M. Hemmi, Caroline C. Kerr, Shaun P. Collin, Craig A. Radford

**Affiliations:** ^1^ School of Agriculture, Biomedicine and Environment La Trobe University Melbourne Victoria Australia; ^2^ CEFE, Univ Montpellier, CNRS, EPHE, IRD Montpellier France; ^3^ CRNL, UCBL, CNRS, INSERM Bron France; ^4^ Australian Centre for Disease Preparedness, Commonwealth Scientific and Industrial Research Organisation Geelong Victoria Australia; ^5^ School of Biological Sciences The University of Western Australia Perth Western Australia Australia; ^6^ Oceans Institute The University of Western Australia Perth Western Australia Australia; ^7^ Institute of Marine Science, Leigh Marine Laboratory The University of Auckland Auckland New Zealand

**Keywords:** biologging, cartilaginous fish, elasmobranch, electroencephalogram, electromyography, electrooculogram, sleep evolution

## Abstract

Sleep is a prominent physiological state observed across the animal kingdom. Yet, for some animals, our ability to identify sleep can be masked by behaviors otherwise associated with being awake, such as for some sharks that must swim continuously to push oxygenated seawater over their gills to breathe. We know that sleep in buccal pumping sharks with clear rest/activity cycles, such as draughtsboard sharks (*Cephaloscyllium isabellum*, Bonnaterre, 1788), manifests as a behavioral shutdown, postural relaxation, reduced responsiveness, and a lowered metabolic rate. However, these features of sleep do not lend themselves well to animals that swim nonstop. In addition to video and accelerometry recordings, we tried to explore the electrophysiological correlates of sleep in draughtsboard sharks using electroencephalography (EEG), electromyography, and electrooculography, while monitoring brain temperature. The seven channels of EEG activity had a surprising level of (apparent) instability when animals were swimming, but also when sleeping. The amount of stable EEG signals was too low for replication within‐ and across individuals. Eye movements were not measurable, owing to instability of the reference electrode. Based on an established behavioral characterization of sleep in draughtsboard sharks, we offer the original finding that muscle tone was strongest during active wakefulness, lower in quietly awake sharks, and lowest in sleeping sharks. We also offer several critical suggestions on how to improve techniques for characterizing sleep electrophysiology in future studies on elasmobranchs, particularly for those that swim continuously. Ultimately, these approaches will provide important insights into the evolutionary confluence of behaviors typically associated with wakefulness and sleep.

## Research Highlights

Some fishes need to swim to breathe, which makes it difficult to identify sleep because the animal is continuously moving. Here, we present relaxed muscle tone as an electrophysiological characteristic of sleep in draughtsboard sharks.

## 1 INTRODUCTION

Animals that respire atmospheric oxygen drown when immersed in water and are deprived of air. Perhaps surprisingly, fishes, including sharks, can also drown, as happens when the flow of oxygenated water over the gills ceases to meet metabolic requirements. Elasmobranchs, a group of cartilaginous fishes of which sharks and rays are prominent members, have two strategies to prevent drowning (Kelly et al., 2020). One approach manipulates the volume (and pressure) of the buccal cavity to actively pump water over the gills, allowing the animal to remain motionless for extended periods of time (buccal pumpers). Another approach uses a pressure gradient, generated by swimming, to ram oxygenated seawater through the open mouth and over the gills (ram ventilators). However, the reliance on continuous swimming might appear to make this ventilatory strategy incompatible with other physiological processes, such as sleep.

In most animals, sleep is a behavioral shutdown marked by quiescence, reduced responsiveness, and rapid arousal to wakefulness with sufficient stimulation (Lesku et al., 2019). Sleep is widespread and perhaps omnipresent across all animals, having been observed even in extant representatives of the earliest invertebrate lineages (Kanaya et al., 2020; Omond et al., 2022). The evolutionary (Libourel et al., 2018; Rattenborg & Ungurean, 2023; Ungurean et al., 2020) and ecological (Kendall‐Bar et al., 2023; Lesku et al., 2012; Lima et al., 2005; Rattenborg et al., 2016; Zaid et al., 2024) persistence of sleep indicates that it must serve nontrivial functions that cannot be accomplished while awake. Indeed, the biological need for sleep is so compelling that natural selection has afforded some animals the ability to sleep concurrent with behaviors often associated with wakefulness. Notably, while sleeping, large herbivores stand (Gravett et al., 2017; Tobler, 1992), ruminants ruminate (Furrer et al., 2024; Ruckebusch, 1972), marine mammals swim (Lyamin et al., 2008) or dive (Kendall‐Bar et al., 2023), and birds fly (Rattenborg et al., 2016). For these reasons, it is likely that fishes, including chondrichthyans and teleosts, have evolved the ability to sleep while swimming (Grainger et al., 2022; Kelly et al., 2019; Kukulya et al., 2016).

To date, research into sleep in sharks has centered around buccal pumping species with clear rest/activity cycles. Quiescent periods have been observed in more than a dozen species of buccal pumping shark in the wild (Kelly et al., 2019). Such anecdotal evidence is suggestive of sleep, but the study of sharks in controlled environments has provided more objective results. Port Jackson sharks (*Heterodontus portusjacksoni*, Meyer, 1793) are benthic and nocturnal, with a pronounced circadian activity rhythm (Kelly et al., 2020). In large tanks, they are active throughout much of the night, and sleep during the day. While asleep, Port Jackson sharks require greater stimulation to arouse them to wakefulness, at which point they rapidly start swimming (Kelly et al., 2021). Epaulette sharks (*Hemiscyllium ocellatum*, Bonnaterre, 1788) are also nocturnal. Sluggish by day, and slow to react, epaulette sharks explore their aquaria at night (Wheeler et al., 2022). Importantly, these sharks also have a low metabolic rate during the day and a 1.7‐fold increase in oxygen uptake at night. These findings suggest that these buccal pumping sharks sleep during the day, since energy savings have been reported in many other sleeping animals, including mammals (Sharma & Kavuru, 2010), fruit flies (Brown et al., 2022; Stahl et al., 2017), and flatworms (Omond et al., 2024).

The species of shark that has received the most attention from sleep scientists is the draughtsboard shark (*Cephaloscyllium isabellum*, Bonnaterre, 1788). Buccal pumping draughtsboard sharks are nocturnal (Kelly et al., 2020). After 5 min of inactivity, they fall asleep, marked by postural relaxation, an increased arousal threshold (Kelly et al., 2021), and reduced metabolic rate (Kelly et al., 2022). Unfortunately, none of these measures are obviously (or easily) applicable to ram ventilating species. Instead, what is needed is an electrophysiological correlate of sleep that can be applied to continuously swimming sharks. In this study, we implanted draughtsboard sharks with electrodes for recording brain activity and temperature, muscle tone, and eye movements to look for an electrophysiological signature of sleep with the potential for applying to other elasmobranchs.

## 2 METHODS

### 2.1 Animals and presurgery housing

Research was conducted in accordance with the guidelines of the New Zealand Code of Ethical Conduct and a University of Auckland animal ethics permit (#001983). Six adult draughtsboard sharks, *Cephaloscyllium isabellum* (four females; weight: 2.2–2.5 kg; total length: 850–915 mm) were sourced as by‐catch from the local fishery off Leigh, New Zealand (36°04′02.3"S, 175°19′24.2E). Upon capture, each shark was placed into an aerated seawater tank and transported to the Leigh Marine Laboratory. Animals were group‐housed in an indoor tank (diameter: 1800 mm, depth: 1600 mm, 4000 L) with natural light conditions (approx. 14 h daylight). Seawater within the tank was filtered (50 µm filter) and temperature‐controlled (16–18°C) via a flow‐through system with full‐volume water changes each hour. In this way, we could maintain natural water quality in terms of temperature, oxygen level, and salinity. Animals were fed within 24 h of collection and every 72 h thereafter on a diet of frozen pilchards and squid, and acclimated to these conditions for at least 2 weeks before surgery. Sharks were monitored daily to ensure good health.

### 2.2 Surgery

**Figure 1:**
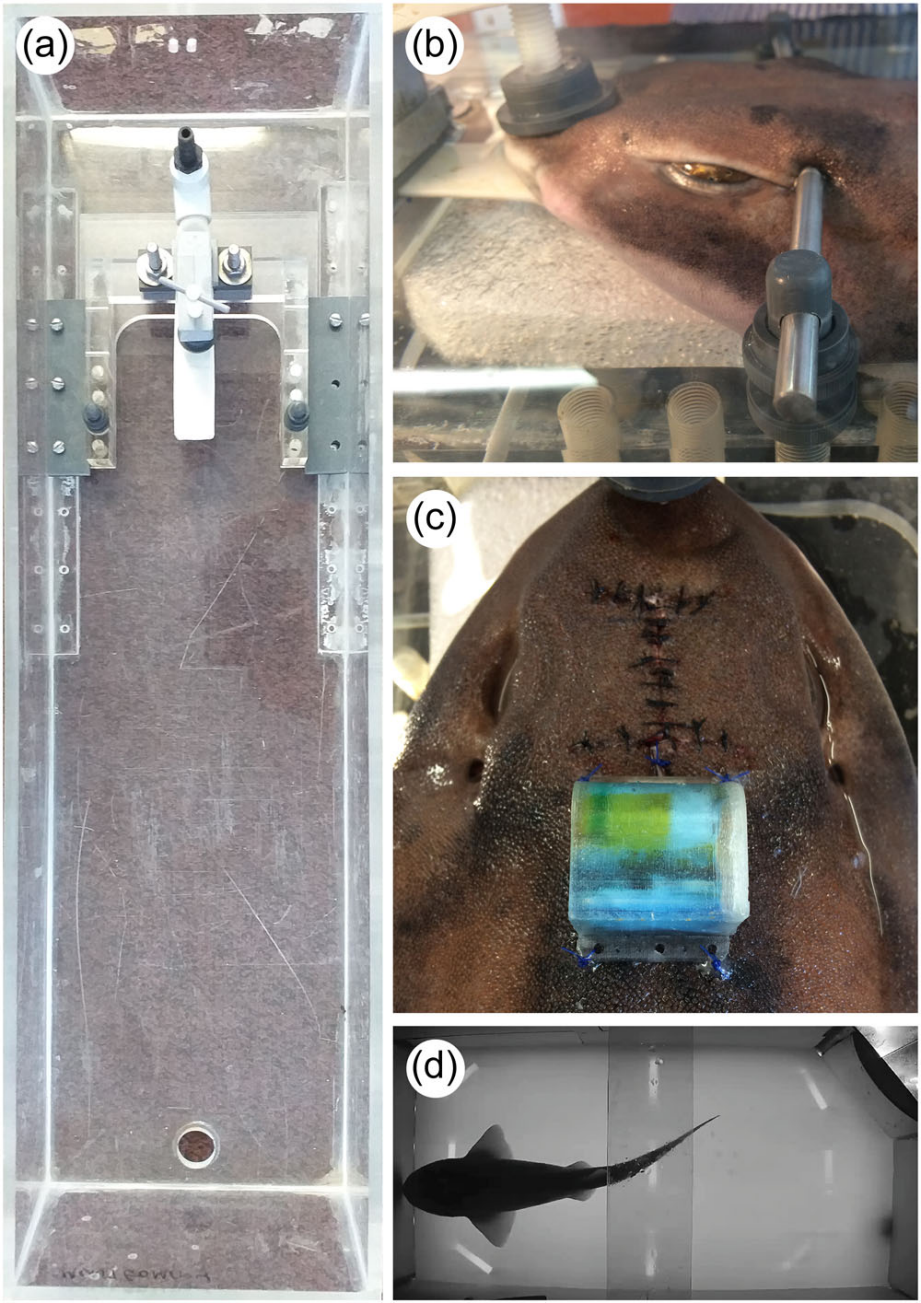
(a) Top‐down view of the acrylic stereotaxic holder. The flattened white tube near the top of the panel delivered buffered MS‐222‐infused seawater into the buccal cavity and out over the gills; the hole at the other end of the surgery tank housed an adjustable pipe for overflow drainage to the sump (when fitted). (b) An anesthetized draughtsboard shark secured in the stereotaxic holder by “ear bars” and nose clamp. (c) At the end of the surgery, the surgical site was closed and the data logger sutured in place. (d) Animals were filmed from above; they appeared as black silhouettes owing to the diffuse, near‐infrared lighting beneath.

Surgical procedures were adapted from those developed in tetrapods (e.g., Johnsson et al., 2022; Lesku, Meyer, et al., 2011; Lesku, Vyssotski, et al., 2011; Libourel et al., 2018, 2023; Shine et al., 2015; Zaid et al., 2024). Sharks were fasted 48 h before anesthesia and inducted into a surgical plane of anesthesia using MS‐222 (tricaine methanesulfonate; 100–120 mg/L) buffered with a 1:2 ratio of sodium bicarbonate. The surgical plane of anesthesia was determined by inactivity, loss of equilibrium, very low respiratory rate, and lack of responsiveness to stimuli. Sharks were then transferred to an acrylic stereotaxic holding device placed inside a surgery tank (length: 920 mm, width: 270 mm, depth: 160 mm) (Figure 1). Water level in the surgery tank was sufficient to cover the gills, but low enough to expose the dorsal surface of the head. Individuals were secured in the stereotaxic holder with a cradle, adjustable “ear bars” to stabilize the chrondrocranium, and a cap providing gentle downward pressure over the rostrum. Throughout the procedure, anesthesia was maintained by the active recirculation of buffered MS‐222‐infused seawater (60–100 mg/L) pumped into the buccal cavity and over the gills.

To expose the chondrocranium, we made a midline incision in the skin (circa 25 mm long) followed by two other perpendicular incisions (25 mm) inscribing a rotated “H” on the head. The skin was deflected to expose the chondrocranium, which was cleaned and dried using 3% hydrogen peroxide. Owing to the absence of a brain atlas, electrode placement was based on (1) detailed examination of similarly‐sized, cadaver draughtsboard sharks and (2) magnetic resonance imaging (MRI) head scans of congener, *C. ventriosum*, Garman, 1880 (http://www.digitalfishlibrary.org). A total of eight holes (0.45 mm diameter) were drilled through the exposed chondrocranium to the level of the dura with a handheld drill (Dremel). Four holes for the silver EEG electrodes (0.0190" diameter; Cat. No. 787500; A‐M Systems) soldered on stainless steel wire (AS633; Cooner Wire Co.) were arranged as a symmetric trapezoid over the left and right regions of the telencephalon. Anterior and posterior holes were drilled 4 and 8 mm caudal to the front edge of the chondrocranium, and 2 and 3 mm lateral of the midline, respectively. Electrodes were held in place with a droplet of cyanoacrylate glue (3 M). Two more holes were drilled over the left‐ and right‐halves of the cerebellum or adjacent optic tectum (circa 18 mm caudal from the anterior telencephalic electrodes, 2 mm lateral of the midline). A seventh hole was drilled to enable a thermistor (Semitec 223Fμ) to be located on the midline, halfway between the telencephalon and cerebellum (i.e., 6 mm caudal from the anterior telencephalic electrodes). The eighth hole was drilled for a control electrode seated off the dura and bathed in cerebrospinal fluid, variably positioned on, or near, the cerebellum. The length of each electrode posed a challenge with respect to the variable depth of the brain surface relative to the overlying chondrocranium. EEG electrodes were 6–11 mm long, depending on the size of the shark and electrode location. Palpating the dura with the electrode to feel resistance was attempted, but the shark brain offered little resistance and we occasionally penetrated the brain (see Section 3). Four pilot holes were drilled for screws. Two stainless steel screws (4 mm length; J.I. Morris Miniature Fasteners) were placed on either side of the cerebellum (4 mm from the midline) and served as EEG reference electrodes. Two brass screws (7 mm length) were placed near the center of the chondrocranium (4 mm off midline) to anchor the dental acrylic to the chondrocranium. Two stainless steel wires, with hooked tip (single braid, 0.0055" diameter; Cat. No. 791000; A‐M Systems) for recording the electromyogram (EMG) were pushed through the skin with a 23 G hypodermic needle to insert the electrodes into the left and right sides of the neck musculature. The same technique was used to place an electrode (Cat. No. 791000; A‐M Systems) over the supraorbital ridge overlying each eye to record the electrooculogram (EOG). All electrode wires, and the thermistor, were consolidated and fed through a polyethylene catheter tube (1 mm diameter) to protect and waterproof the electrodes, before attaching to the data logger. The surgical site was sealed with dental acrylic (Paladur, Kulzer GmbH) and the catheter tube was fixed in place with cyanoacrylate glue. The logger was sutured to the skin 30 mm caudal to the surgical site using 2 mm nylon suture (Flexocrin 2/0 DS19). Once the surgery was completed, the animal was transferred to an experimental tank at which time continuous video and electrophysiological recording began. Animals began swimming within 10 min. The entire surgery from induction to recovery took 4–5 h. All sharks were fed within 24 h of postoperative recovery.

### 2.3 Video and electrophysiological recordings

For the recordings, animals were housed individually in experimental tanks (length: 1200 mm, width: 600 mm, depth: 540 mm, volume: 390 L) and maintained on a 12:12 light:dark photoperiod provided by aquarium LED lights (Zetlight Lancia ZP4000 Marine Light, 1200 mm, 46 W), suspended 1 m over each tank. Lights began to turn on at 0700 h, and off at 1830 h, to emulate a 30‐min dawn and dusk, comparable to twilight (10.8 lux). Light intensity during the day was 80 lux, comparable to light levels 5 m underwater on an overcast day, and 0 lux at night. Immediately under the light was a polytetrafluoroethylene sheet (1 mm thick), which acted as a diffuser to reduce reflection on the seawater. For filming, near‐infrared LED strip lights (850 nm single chip, 120 LEDs 9.6 W/m, 12 V DC), using a wavelength undetectable by this species (Hart et al., 2011), were fixed in parallel rows equidistant from one another (100 mm apart) along the length of the floor underneath each tank. A diffuser (3 mm thick) was placed over the LED lights to provide homogenous background lighting to the cameras above. The animals were filmed using an overhead infrared sensitive camera (Altronics, 4.0 megapixel, weatherproof vari‐focal IP PoE) fitted with a red gel filter (Lee Filters, Pop Red).

Electrophysiological recordings were made using a custom‐built data logger (20 × 10 x 10 mm, 15 g). The logger was encased in an acrylic shell filled with waterproofing gel (Raytech Bi‐Component Polymer Gel). Recordings commenced 30 min before the start of each surgery, which were intended to run continuously for 6 days. We attempted to record seven referential EEG channels (four telencephalon, two cerebellar/optic tectum, one control), two differential channels (one EMG and one EOG), one thermistor, and each of the three cardinal axes of the accelerometer. Only the EMG, thermistor, and accelerometer would ultimately have long‐term stability; only half the loggers collected data (see Section 3). After 1 week, sharks were euthanized with an overdose of buffered MS‐222 and decapitated; heads were immersion fixed in 4% paraformaldehyde in 0.1 M phosphate buffer for bioimaging to assess electrode placement. After a minimum fixation period of 8 weeks, shark heads were shipped to Melbourne, Australia, and scanned at Monash University Biomedical Imaging by a Siemens Somatom GoUp™ computed tomography (CT) scanner. Images were segmented manually using Avizo 7.0 (Thermo Fisher Scientific, Pty. Ltd.).

### 2.4 Analyses

Video footage was processed by the software Digi (Hemmi & Tomsic, 2015). Shark position was tracked at two frames‐per‐second by comparing each frame with a background image that did not show a shark. After applying a threshold to the difference image, blob analysis in MATLAB (The MathWorks, Inc.) was used to identify independent, labeled regions. This allowed us to identify the size and position of each region (blob). The largest region closest to the shark's position in the previous image was taken as its new location. Data were filtered using a boxcar filter of 1 min (120 frames) and subsampled to one sample per minute. Video data was then synchronized with electrophysiological data, which was analyzed using SlipAnalysis, a custom MATLAB program to visualize and analyze electrophysiological and behavioral data. We obtained signals from three of six sharks. The first 6‐h of each recording was excluded to avoid any latent effects of the MS‐222. For two sharks, we logged 6 days of recordings, but only a single day for the third shark. The accelerometry signals were used to determine the state of the sharks (i.e., active or quiet wakefulness, or sleep). Specifically, states were extracted by calculating the norm of acceleration over the three axes. The signal was detrended by a moving window of 1 min and redressed; it was then smoothed by a sliding median filter of 10 s length. In doing so, we could characterize active sharks (acceleration >0.01 G for more than 15 s), quietly awake sharks (acceleration <0.01 G for the first 15–180 s following the active state), or sleeping sharks (acceleration <0.01 G for more than 15 min). To avoid ambiguous transition states, we excluded the first and last 5 min of each sleep bout. The significance of differences observed in the amount of activity and muscle tone, between states, was assessed using a paired *t*‐test after Gaussian normalization in MATLAB.

## 3 RESULTS

**Figure 2:**
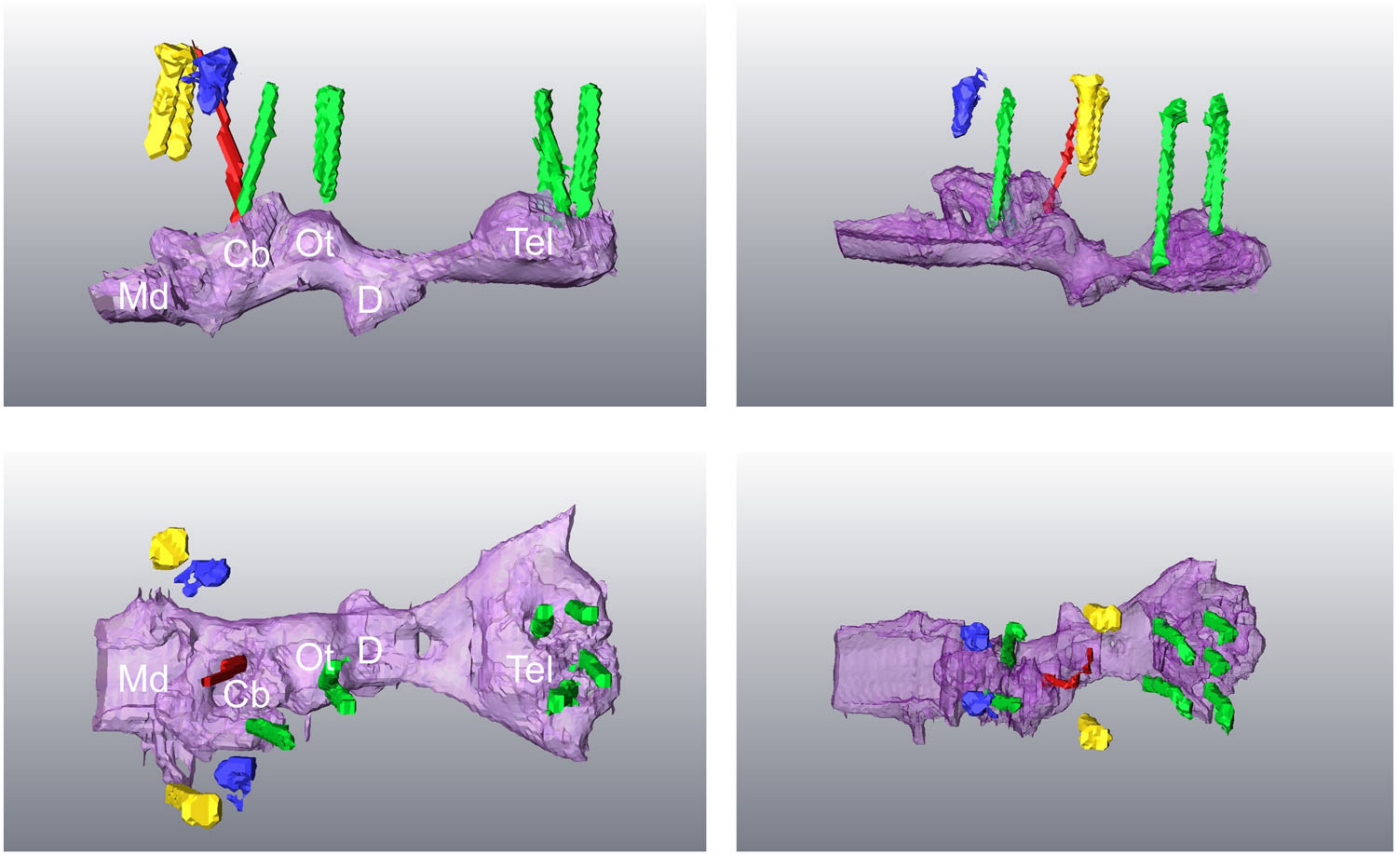
Segmented images from computed tomography (CT) scan for two of the three experimental draughtsboard shark brains from lateral (*top row*) and dorsal (*bottom row*) viewpoints. Imaging of the third shark was not successful. Shown are referential electrodes for the telencephalon, and cerebellum or optic tectum (*green*), reference screws (*blue*), thermistor (*red*), and anchor screws (*yellow*). In all images, the brainstem is on the left. Owing to tissue shrinkage during fixation, it is possible that electrodes seen above the brain here, were touching the brain in vivo. Cb, cerebellum; D, diencephalon; Md, medulla oblongata; Ot, optic tectum; Tel, telencephalon.

The most basic outcome is that we were successful in targeting the telencephalon and cerebellum or optic tectum (Figure 2). However, finer electrode placement was unintentionally variable; notably, whether each electrode sat above, on, or within the surface layers of the brain. With this context made clear, we next consider the electroencephalogram (EEG) recordings, and then move onto the electrooculogram (EOG), and electromyogram (EMG) outcomes.

**Figure 3:**
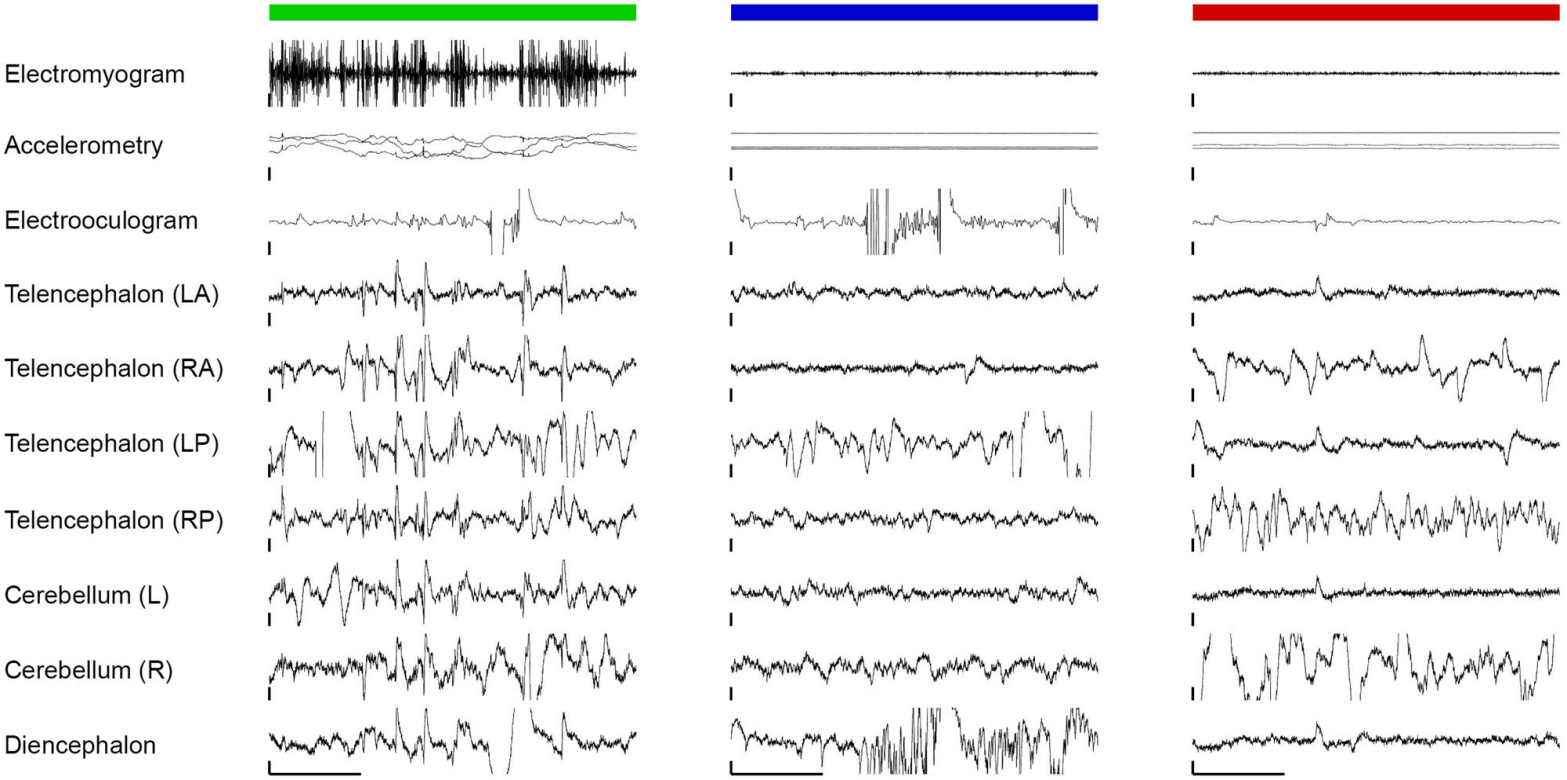
Exemplar raw‐data electrophysiological traces from a shark actively awake (*green*), quietly awake (*blue*), and asleep (*red*). Signals reflect our attempt to record skeletomuscular tone (electromyogram; high‐pass filter: 10 Hz, scale: 40 µV), movement (accelerometry in *X*, *Y*, and *Z* axes; scale: 1 G), ocular activity (electrooculogram; low‐pass filter: 10 Hz, scale: 50 µV) followed by signals from the left (L) or right (R) anterior (A) or posterior (P) telencephalon, cerebellum, and diencephalon (all scaled at 20 µV). Using the computed tomography (CT) scans, it appears as though the diencephalon and telencephalon electrodes were in contact with the surface of the brain, but the RP electrode penetrated the dura. Both cerebellar electrodes (unintentionally) also penetrated the dura. These traces show that brain activity was obscured in swimming sharks. Yet, even in stationary sharks, signals were unstable. *X*‐axis scale bars denote 5 s, such that each trace is 20 s.

We examined signals based on whether the animal was moving (active wakefulness), inactive for less than 3 min (quiet wakefulness) or inactive at least 5 min (asleep); the latter is a state associated with increased arousal threshold (Kelly et al., 2021) and reduced metabolic rate (Kelly et al., 2022). Not surprisingly, sharks that were quietly awake moved less than those actively awake, and more than those that were asleep. Electroencephalographic signals from moving animals were often contaminated by high amplitude artifacts, which prevented a true comparison with other states (Figure 3). Furthermore, it is unclear whether variation in the signals reflect real physiological differences between electrode channels and states, or intermittent contact between the electrode and tissue, and/or tissue trauma during implantation. Accordingly, within a state, including motionless sleep, there was substantial differences in signal amplitude and frequency at different electrode sites at the same time, and during recordings at the same electrode site at different times, suggestive of a nonphysiological origin of signals. That said, on some occasions we obtained stable signals from moving sharks (Video S1), yet randomly and too infrequently for a meaningful analysis.

**Figure 4:**
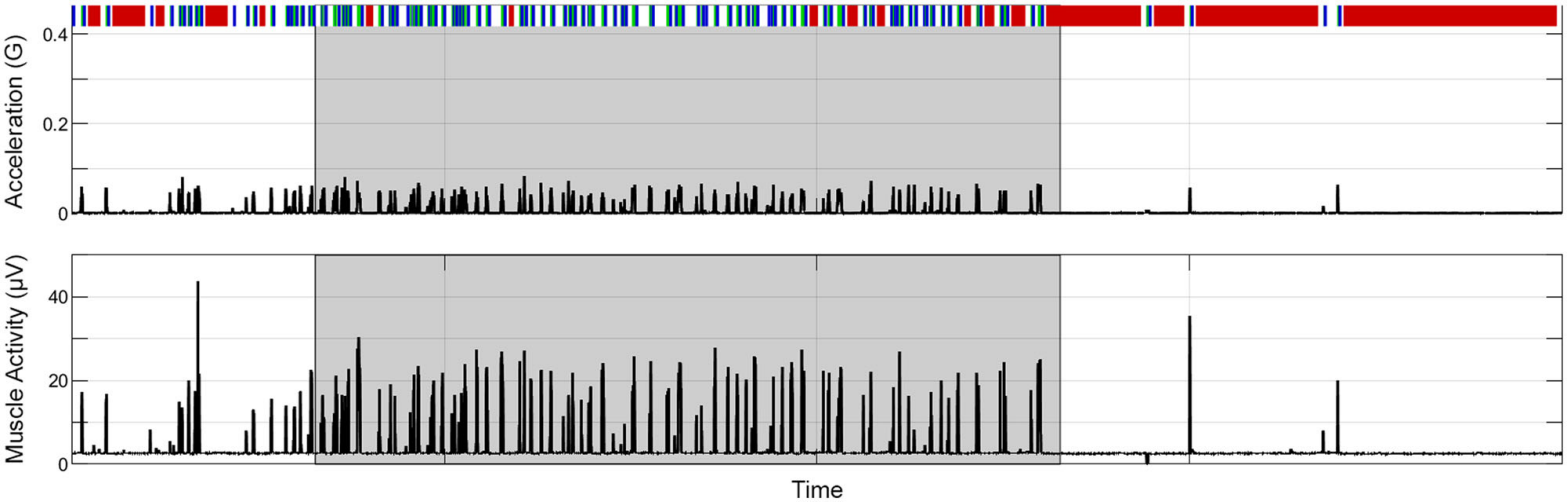
Processed accelerometry and electromyogram signals over a representative 24‐h day from one shark housed under a 12:12 light:dark photoperiod. Gray shading denotes the 12‐h night, starting at 1900 h. These plots show the strong alignment between movement and muscle tone during active (*green*) and quiet (*blue*) wakefulness, and sleep (*red*).

**Figure 5:**
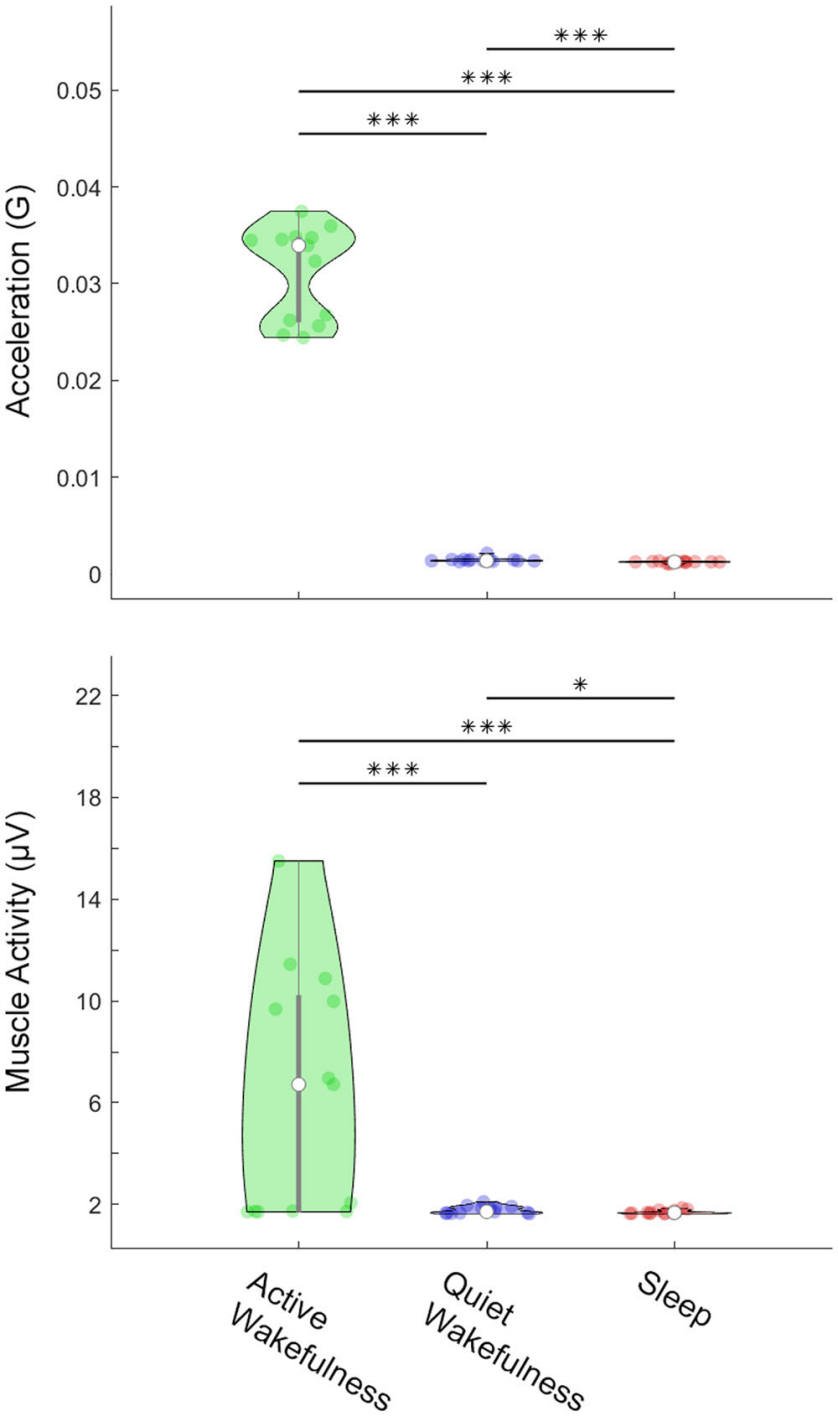
The degree to which sharks moved (*top*) and their level of muscle tone (*bottom*) depended on whether they were actively or quietly awake, or asleep. Muscle tone was highest during active wakefulness and lowest during sleep. Datapoints represent 24‐h means per shark, with 1–6 days per shark. Significance is marked by asterisks (*p* = 0.03*; *p* < 0.001***).

What of temperature, eye movement, and muscle tone? Brain temperature was numerically the same as the temperature of the seawater across the recording and across states (data not shown). Despite the differential measure of the EOG, we failed to identify with certainty any eye movements, as transient artifacts were present in the signal likely owing to fluctuations in the reference. We did successfully measure muscle tone from freely behaving sharks, as the two muscle electrodes were closer than the two ocular electrodes. Muscle tone was strongest during active wakefulness, lower during quiet wakefulness, and lowest during sleep (Figures 4 and 5).

## 4 DISCUSSION

To our knowledge, this is the first electrophysiological investigation into the correlates of sleep in any cartilaginous fish, and one of a handful for bony fishes (Kelly et al., 2019). Previous recordings of brain activity from sharks focused, not on sleep, but measuring sensory evoked potentials from anesthetized or awake animals (Bullock & Corwin, 1979; Casper & Mann, 2006, 2007, 2009; Cohen et al., 1973; Gilbert et al., 1964; Nieder, Gibbs, et al., 2023; Nieder, Rapson, et al., 2023; Platt et al., 1974). The sharks used in our study awoke and swam after surgery, and ate within 24 h. For three of the six sharks, the logger, and its accelerometer, also operated while submerged in seawater. We were successful in placing electrodes on, or in, the telencephalon and cerebellum or optic tectum. Signals from the muscle were ultimately the most informative. These procedural successes were sadly accompanied by disappointments in data quality, as detailed below.

By way of background and comparison, electrophysiological data on sleeping bony fishes is also scarce. The catfish (*Ictalurus nebulosus*, Lesueur, 1819) shows EEG slow‐wave activity and spiking from the forebrain and midbrain during restfulness, which disappears on arousal to wakefulness (Karmanova & Belich, 1983; Karmanova et al., 1981). Tench (*Tinca tinca*, Linnaeus, 1758) in a quiescent state have lower muscle tone and slower respiratory rate, but otherwise show no changes in EEG activity (Peyrethon & Dusan‐Peyrethon, 1967). With respect to brain activity in the draughtsboard shark, we unfortunately have little new insights to offer. Even in sleeping sharks, in which movement was absent, brain activity was highly variable at one, and also between, electrodes. This variability is because the brain is floating, and perhaps moving, within the endocranial cavity against the electrodes. As a result, we could not identify any repeatable pattern across days and individuals. This variability is reminiscent of early uncertainties in the nonavian reptile sleep literature 50 years ago. For example, reports on the electrophysiological correlates of sleep in caiman (*Caiman sclerops*, Schneider, 1801) were diverse and included evidence for nonREM sleep‐like slow‐waves (Meglasson & Huggins, 1979; Warner & Huggins, 1978), yet also no slow‐waves and instead high‐voltage sharp waves (Flanigan et al., 1973). Similarly, there were reports for (Peyrethon & Dusan‐Peyrethon, 1969) and against (Flanigan et al., 1973) REM sleep in caiman. No signs of sleep, either behaviorally or electrophysiologically, were observed in the American alligator (*Alligator mississippiensis*, Daudin, 1802) (Van Twyver, 1973). Similar inconsistencies were reported also in lizards (reviewed in Libourel & Herrel, 2016). Only through careful and systematic study over the last decade has the unfolding story of sleeping reptiles been told (Fenk et al., 2023; Libourel et al., 2018; Norimoto et al., 2020; Shein‐Idelson et al., 2016; Tisdale et al., 2018). A similar effort should be initiated on sharks.

Even in the absence of brain activity correlates for sleep in buccal pumping draughtsboard sharks, we have learned two valuable things. Draughtsboard sharks have the best characterized sleep state of all cartilaginous fishes thus far. When these sharks fall asleep, they are restful (Kelly et al., 2020) and adopt a recumbent (flat) body posture (Kelly et al., 2022). After 5 min of inactivity, they show reduced responsiveness to weak electrical stimulation (Kelly et al., 2021), and a lower oxygen consumption rate (Kelly et al., 2022). To this inventory, we can now add that muscle tone is lowest in sleeping draughtsboard sharks. Sadly, we were not able to extract any eye movements from the EOG; yet doing so may add an important characteristic for identifying sleep in continuously swimming sharks, as it has been in reptiles (Libourel et al., 2018). The second value comes from a new‐found appreciation for what not to do. The lackluster stability of EEG signals arose from three factors. First, we relied on a single reference electrode, such that fluctuations in the reference impacted all channels. Second, unlike birds and mammals whose brain sits flush against the skull, the brain of the draughtsboard shark does not contact the dorsal cranium, but instead is bathed in cerebrospinal fluid with a variable gap. Thus, some electrodes had intermittent contact with the brain. Third, owing to across‐species variation in the thickness of the dura (Kinaci et al., 2020), which feels thin in sharks, palpating the tissue with an electrode was ambiguous, and potentially destructive. Positioning the electrodes without a standardized stereotactic atlas was challenging, as long electrodes inserted through a relatively thin chondrocranium meant that electrodes were not always seated where they were intended. For the future, we would recommend (1) having a stable reference electrode near to the recording electrode, (2) improve signal stability by recording local field potentials within the brain, rather than electrodes on the surface of the brain, (3) selecting a species for whom a brain atlas exists and (4) whose brain contacts the dorsal cranium, such as a (dorsoventrally flattened) ray, and (5) including ample nonneural measures that might correlate with sleep, including eye movements, but also heart and respiratory rate, that might be informative for use on ram ventilating sharks.

Presently, we have only glimpses of what sleep might look like in ram ventilating sharks. An adult female white shark was filmed by an autonomous underwater vehicle in the near‐shore waters off Guadalupe Island, Mexico (Kukulya et al., 2016). The submersible was equipped with five video cameras; lights in the nose of the submersible provided illumination. At night, the shark swam slowly, without changes in speed or direction, and swam into the underwater current with her mouth agape. Given the serendipitous nature of this observation, it is unclear how common, or for how long, this behavior persists among white sharks, or whether is exists in other ram ventilating species. Nonetheless, leisurely swimming into the current might allow ram ventilating species to sleep and breathe due to the passive movement of water across the gills. Another compelling behavior has been observed in juvenile white sharks off the east‐coast of Australia (Grainger et al., 2022). To the pectoral fin of eight white sharks (equal sexes) was attached a tag containing an accelerometer, gyroscope, magnetometer, depth sensor, and video camera. Sharks were observed slowly swimming, and indeed could be considered, rheotaxically, almost stationary, by swimming into a current. Most compelling is that the sharks swam slowly in a circular pattern during the daytime, alternating between clockwise and counter‐clockwise rotations, taking 1–2 min per rotation. Periods of circular swimming ranged between 20 min to 4.7 h. The sharks appeared to be uninterested (or unaware) of potential prey swimming nearby. This tortuous pattern of swimming in juvenile white sharks is reminiscent of the cork‐screw patterns traced by northern elephant seals (*Mirounga angustirostris*, Gill, 1866) sleeping during oceanic dives (Kendall‐Bar et al., 2023) and the microsleeps of great frigatebirds (*Fregata minor*, Gmelin, 1789) as they circularly soar on thermals (Rattenborg et al., 2016; see also Libourel et al., 2023). Moreover, ruminants, such as reindeer (*Rangifer tarandus*, Linnaeus, 1758) blur the line between behaviors typically associated with wake and sleep. They generate sleep‐like brain activity while chewing their cud and dissipate sleep pressure as a result, indicating that they achieve the neurological benefits of sleep while ruminating (Furrer et al., 2024). Whether any of these behaviors of white sharks reflect sleep, or something else, is unknown. In the future, recording a combination of parameters that includes local field potentials, muscle tone, eye movements, heart and breathing rate would improve our understanding of when, and how, ram ventilating sharks sleep.

## CONFLICT OF INTEREST STATEMENT

The authors declare no conflict of interest.

## Supporting information

Supporting information.

## Data Availability

The data that support the findings of this study are available from the corresponding author upon reasonable request.
